# The Overexpression of IQGAP1 and β-Catenin Is Associated with Tumor Progression in Hepatocellular Carcinoma *In Vitro* and *In Vivo*

**DOI:** 10.1371/journal.pone.0133770

**Published:** 2015-08-07

**Authors:** Xuewen Jin, Yuling Liu, Jingjing Liu, Weiliang Lu, Ziwei Liang, Dan Zhang, Gang Liu, Hongxia Zhu, Ningzhi Xu, Shufang Liang

**Affiliations:** 1 State Key Laboratory of Biotherapy and Cancer Center/Collaborative Innovation Center for Biotherapy, West China Hospital, Sichuan University, No.17, 3rd Section of People's South Road, Chengdu, 610041, P. R. China; 2 Department of Rheumatology, West China Hospital, Sichuan University, Chengdu, 610041, Sichuan, P. R. China; 3 Laboratory of Cell and Molecular Biology & State Key Laboratory of Molecular Oncology, Cancer Institute & Cancer Hospital, Chinese Academy of Medical Sciences, Beijing,100034, P. R. China; University of Alabama at Birmingham, UNITED STATES

## Abstract

The IQ-domain GTPase-activating protein 1 (IQGAP1) is a multifunctional scaffold protein, which interacts with diverse proteins to regulate cell adhesion and cell migration. The abnormal expression of IQGAP1 widely exists in many cancers, but biological roles of IQGAP1 cooperation with its interacting proteins to involve in tumorigenesis remain to clarify. In this study, we have found that IQGAP1 interacts with β-catenin and regulates β-catenin expression in hepatocellular carcinoma (HCC) cells. The expression levels of IQGAP1 and β-catenin and their associations have a positive correlation with cell metastasis ability in several HCC cell lines. The up-regulation of IQGAP1 and β-catenin improves cell proliferation and migration ability of HCC cells, whereas the knockdown of IQGAP1 by small interfering RNA can decrease β-catenin expression, which results in the reduction of cell proliferation and migration ability *in vitro*. In addition, a significantly higher expression of IQGAP1 and β-catenin also usually exists in human HCC tissues, especially their overexpression is clinicopathologically associated with tumor malignancy. Generally the overexpression and interactions of IQGAP1 and β-catenin contribute to HCC progression by promoting cell proliferation and migration.

## Introduction

Hepatocellular carcinoma (HCC) is one of the most common cancers in the world, and its prognosis depends on the tendency to metastasize [1, 2]. Although significant advances have been made in the treatment of metastatic HCC, a rapid recurrence and poor survival of HCC is still a challenging problem. Therefore, a thorough understanding of the molecular mechanisms of HCC metastasis is urgently required to identify effective diagnosis and therapeutic targets, which facilitates early treatment of HCC patients with a high risk of metastasis to improve the survival[2].

The IQ-domain GTPase-activating proteins 1 (IQGAP1) is a scaffold protein that regulates distinct cellular processes including cell adhesion, cell migration, extracellular signals through interacting with numerous proteins [3, 4]. Multiple studies have shown that IQGAP1 is up-regulated in many human malignancies, such as lung cancer[5], ovarian cancer[6], colon cancer[7], breast cancer[8, 9], melanoma[10] and HCC[11]. And IQGAP1 plays a critical role in cancer cell invasion. The overexpression of IQGAP1 in colon cancer cell correlates with poor prognosis [12], and cell motility was increased by overexpressing IQGAP1 in breast cancer [13]. IQGAP1 can directly interact with CDC42 and Rac1 to regulate E-cadherin mediated cell-cell adhesion[14]. IQGAP1 can also regulate nuclear localization of β-catenin, which also involves in cell transformation and migration in WNT signaling [15]. However, the biological associations and effects between IQGAP1 with β-catenin are not discovered in HCC.

In this study, we focused the protein expression level of IQGAP1 and β-catenin in HCC cells and tissues and their associations with tumor malignancy degree, as well as their roles in cell proliferation and migration ability. Our findings have discovered that the expression of β-catenin can be affected by the IQGAP1 level, and their overexpression and interaction improve cell proliferation and migration ability in HCC progressing processes.

## Methods

### Cell culture

Human HCC cell lines HuH7 and HepG2 were obtained from the American Type Culture Collection. Human HCC cell lines MHCC-97H and MHCC-97L were established in the Liver Cancer Institute of Fudan University[16], and these cells were generously endowed for our research. MHCC-97H and MHCC-97L cells were originally established from the same parent cell line MHCC-97, with orthotopic inoculations of MHCC-97H and MHCC-97L to recipient nude mice respectively, with a spontaneous pulmonary metastasis occurred in 100% and 40%[16]. The normal liver cell line LO2 was ordered from Cell Bank of Shanghai Institute of Cell Biology, Chinese Academy of Sciences. All these cells were maintained in the Dulbecco's Modified Eagle's Medium (DMEM) supplemented with 10% fetal bovine serum (FBS) (16000–044, Gibco), 100 U/ml penniciline, and 100ug/ml streptomycin. Cells were incubated in a 37°C incubator with 5% CO_2_ and 95% air.

### HCC samples

Thirty-three pairs of HCC tissues (HCTs) and patients’ autologous para-cancerous liver tissues (PLTs) were surgically resected to collect in West China Hospital, Sichuan University (Chengdu, P. R. China). Informed written consents were obtained from the patients. This project was approved by the Institutional Ethics Committee of State Key Laboratory of Biotherapy, West China Hospital of Sichuan University.

All tissues were immediately frozen after surgical operation in liquid nitrogen prior to experiments. The patients’ gender, age and histological type of tumor differentiation were obtained from the surgical and pathological records. And the histological type of tumor differentiation was classified into high differentiation group and low differentiation group[17]. Each case was identified through pathologic biopsy.

### RNA interference

Small interfering RNA (siRNA) against IQGAP1 were synthesized as the following sequences [18]: IQGAP1, 5’-UUA UCG CCC AGA AAC AUC UUG UUG G-3’; Negative control oligonucleotides (NC) were 5’-UUC UCC GAA CGU GUC ACG U-3’. And the RNA interference experiments were performed with the reagent Lipofectamine 2000 following the manufacturer’s protocols.

### Plasmids and cell transfection

The IQGAP1 cDNA (GenBank gi57242794) was cloned into pCMV6 plasmids by PCR-based cloning method, and the recombinant plasmid pFlag-IQGAP1 was obtained. The β-catenin cDNA (GenBank gi 148233337) was cloned into pEZ-M13 plasmids by PCR, and the recombinant plasmid was named pFlag-β-catenin. Cell transfections of pFlag-IQGAP1 plasmids were performed using Lipofectamine LTX reagent (15338030, Life Technologies), and the transfections of pFlag-β-catenin plasmids were performed with the reagent Lipofectamine 2000 (11668–019, Life Technologies) following the manufacturer’s protocols. The amount of transfected plasmids was 2.5μg for each well of a 6-well plate, and the IQGAP1-specific siRNA was used 100nM for one well of a 6-well plate. In the co-transfection of pFlag-β-catenin plasmids and IQGAP1-specific siRNA, 2.5μg plasmids and 100 nM siRNA was used for one well of a 6-well plate at the same time.

### RNA extraction and real-time PCR

Total RNA was extracted using Trizol reagent (Cat. #15596–026, Invitrogen). The first-strand cDNA was generated using a cDNA synthesis kit (Cat. #170–8891, Bio-Rad). The real-time PCR was performed to measure the relative expression using the Supermix-Bio-Rad kit (Cat. #172–5261, Bio-Rad). The GAPDH mRNA level was taken as a comparison base for the target RNA expression. The relative RNA expression was calculated with the comparative CT method, which was normalized to the internal references. The primers were designed as follows. IQGAP1: forward primer 5′-TCC ATT ACT TAG GAA AGA GTG GAA ACT-3′ and reverse primer 5′-CAA ACA CCA AAG CTT ACA ATA TAG TAC TGC-3′; forward primer 5′-AAG CTC TTA CAC CCA CCA TCC-3′ and reverse primer 5′-GTG CAT GAT TTG CGG GAC AAA-3′ for β-catenin; forward primer 5′-CGT CTC CAC ACA TCA GCA CAA-3′ and reverse primer 5′-TCT TGG CAG CAG GAT AGT CCT T-3′ for c-myc; forward primer 5′-TGT TCG TGG CCT CTA AGA TGA AG-3′ and reverse primer 5′-AGG TTC CAC TTG AGC TTG TTC AC-3′ for Cyclin D1; forward primer 5′- GGG CCA CTT TAA AGA GCA G -3′ and reverse primer 5′- CCT TCA TAC ATC GGG AGC A -3′ for Axin2; forward primer 5′- TGG AAG GAC TCA TGA CCA CA-3′ and reverse primer 5′- TTC AGC TCA GGG ATG ACC TT-3′ for GAPDH.

### Luciferase reporter gene assay

The promoter fragments of β-catenin (from -1018 to +241) were cloned into pGL3 Basic luciferase reporter plasmid (Promega), and the pGL3-catenin plasmid was obtained. HepG2 cells were transfected with 0.5μg or 2μg pFlag-IQGAP1 plasmids to incubate for 24 hours, then transfected with 1μg pGL3-catenin plasmids or the empty pGL3-basic plasmids to incubate for 24 hours. Cells were harvested to assess the luciferase activity according to a commercial Dual-luciferase reporter assay system (Cat. #E1910, Promega). 100 ng pRL-TK (Promega) renilla luciferase was co-transfected in each sample as an internal control for transfection efficiency. All experiments were performed in triplicate from independent cell cultures.

### Transwell migration assay

Cell migration ability was assayed based on a transwell chamber apparatus (Millipore). After being transfected with pFlag-IQGAP1 plasmids or IQGAP1-specific siRNA for 48h, cells were seeded in the upper chamber of a transwell, and the bottom of the chambers was filled with 600μl of DMEM containing 10% FBS. 1×10^4^ cells in 200ul serum-free DMEM were cultured in the upper wells for 24 hours. The migrated cells were fixed and stained with 1% crystal violet. Images were captured using an inverted microscope (Olympus), and the migrated cells were counted manually. The percentage of migrative cells on the condition of IQGAP1 overexpression or knockdown was calculated by comparison with the control transfection with the empty plasmids or non-targeting siRNA.

### Cell viability

Cell viability was measured using MTT assay. After IQGAP1 overexpression or knockdown for 48h, 5×10^3^ cells/well were seeded in one 96-well plate in DMEM supplemented with 10% FBS to incubate for 24h. And 20ul of MTT solution (5 mg/ml, Sigma) was added to each well to incubate for 2–4 h at 37°C, the formazan crystals were dissolved with 150ul Dimethyl Sulfoxide (Sigma). Absorbance was determined at 570 nm on Multiskan MK3 (Thermo Scientific) immediately. Each assay was separately performed for three replicates and all experiments were repeated at least three times. The cell viability percentage was estimated as follows.

Cell viability (%) = absorption test/ absorption control ×100%

Meanwhile, in order to discriminate if cell growth inhibition was only due to changes in cell proliferation or due to cell death, cells under IQGAP1-specific siRNA treatment were detected cell death by flow cytometry analysis using BD Bioscience Annexin V/PI staining kit (556547, BD Biosciences). After cells were transfected with siRNA for 48h, cells were harvested to stain with 5 μl Annexin V-FITC and 5 μl propidium iodide (PI) for 15 min at room temperature in the dark. Then cell suspensions were immediately evaluated by flow cytometry (NovoCyte, ACEA Biosciences). The apoptotic cells were analyzed based on the percentage of Annexin V-positive cells, and the necrotic cells were analyzed based on the percentage of Annexin V-negative and PI-positive cells. Cell death included the apoptotic cells and necrotic cells.

### Immunoprecipitation

HepG2 cells were harvested to dissolve with a co-immunoprecipitation (co-IP) buffer containing 20mM Tris (pH7.5), 150mM NaCl, 1% Triton X-100 and protease inhibitor cocktail (05892970001 Roche). 1–2μg of anti-IQGAP1 antibodies (SC-81906, Santa Cruz Biotechnology) was incubated with 50μl slurry of protein A+G conjugated sepharose (P2012 Beyotime) to pull down protein complexes from cellular lysates (500μg per sample). And 1–2μg of mouse IgG (A7028 Beyotime) was taken as a control. After washing 4 times, the protein complexes were eluted with SDS-PAGE sample-loading buffer (P0015 Beyotime) and loaded onto PAGE gel for western blot detection.

### Western blot

Cells were lysed with RIPA buffer (50 mM Tris base,1.0 mM EDTA, 150 mM NaCl, 0.1% SDS, 1% Triton X-100, 1% sodium deoxycholate, 1 mM PMSF). For protein subfractionation, nuclear and cytoplasmic proteins were respectively extracted from HepG2 cells transfected with pFlag-IQGAP1 plasmids by using NE-PER Nuclear and Cytoplasmic Extraction Reagents (#78833, Pierce). Proteins were separated by 10% SDS-PAGE electrophoresis, and transferred from the gel onto the PVDF membrane (Amersham Biosciences) to detect protein expression level. The PVDF membrane was respectively incubate with anti-IQGAP1 (ab133490, Abcam) and anti-β-catenin monoclonal antibody at a dilution of 1:1000 at 4°C overnight, followed by three 15-min washes in phosphate-buffered saline within 0.1% Tween-20. The membranes were then incubated with HRP-conjugated secondary antibodies at 37°C for 60 min. Detection was performed with Western blot reagent ECL (Amersham Biosciences). The GAPDH detection against its rabbit monoclonal anti-GAPDH antibodies with being diluted 1:5000 [EPR16891] (ab181602, Abcam) was taken as a control. The nuclear protein LMNB1, as a nuclear housekeeping control, was detected against anti-LMNB1 antibodies with a dilution 1:1000 [ag3631] (12987-1-AP, Proteintech). The Image J software was used to quantify the Western blot result.

### Immunohistochemistry

The HCTs, PLTs were paraffin-embedded and cut into sections with 5μm-thickness for hematoxylin-eosin and immunohistochemistry (IHC) analysis mainly according to our previous protocols [19]. The anti-IQGAP1 antibody was diluted 1:500 (ab133490, Abcam), and the anti-β-catenin antibody was used a dilution of 1:400 (ab32572, Abcam). The second antibody was a biotinylated IgG to incubate 40 min at 37°C. Finally, the tissue slices were visualized by the 3, 3-diaminobenzidine solution and counterstained with hematoxylin. Substitution of the primary antibody with phosphate-buffered saline was served as a control for IHC.

The IHC scoring analysis of tissue slices was evaluated using light microscopy by two pathologists blinded for patient outcome to minimize variability. The estimated percentage of staining was determined by calculating average staining cells of 3–4 microscopic fields under 400-fold magnification. According to general evaluation standards[20], the proportion of positive staining cells was scored with 0–4. The positive staining below 5% was defined as 0, 6–25% positive staining was set as 1, and 26–50% positive staining was scored 2. The staining with 51–75% was defined 3, and staining ratio over 75% was scored 4. Meanwhile, staining intensity was graded 4 levels as follows: 0, negative; 1, weak; 2, moderate; 3, strong. Meanwhile, the immunoreactivity score for each slice, ranging from 0 to 12, was measured as immunostaining intensity multiplied by the percentage of positive cells. The score of 0 was defined as a negative expression (**-**); scores of 1–3 were accepted as a weakly positive expression (**+**), while 4–6 scores were defined as a moderately positive expression (**++**), and 7–12 scores were accepted as a strongly positive expression (**+++**), namely overexpression (scoring > 7).

### Statistical analysis

All data were determined as mean ± standard error of the mean (SEM). Comparisons between two groups were performed by Student’s t test. Differences among multiple groups were assessed by one-way ANOVA analysis. And Spearman correlation was used to analyze the correlation of data. *p*<0.05 was considered to be statistically significant.

## Results

### IQGAP1 interacts with β-catenin in HCC cells

**Fig 1:**
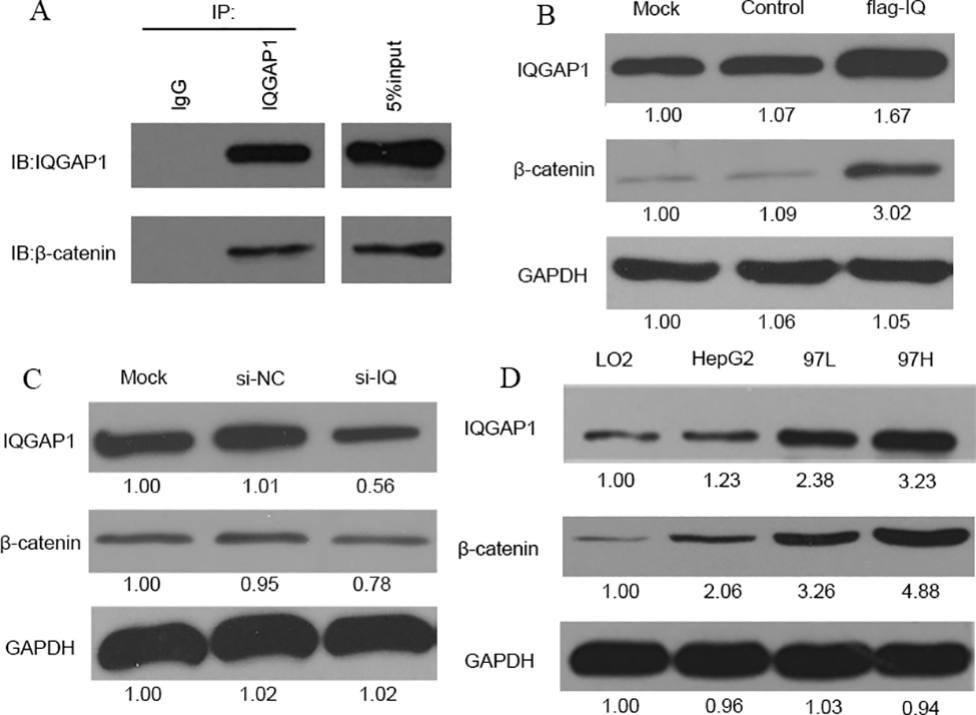
IQGAP1 interacts with β-catenin and regulates β-catenin expression. (A) Endogenous IQGAP1 interacts with β-catenin in HepG2 cells. 500ug of HepG2 cell lysate was immunoprecipitated with anti-IQGAP1 antibodies, and bound proteins were analyzed by immunoblotting with the indicated antibodies. 5% of total lysate was loaded as a control (input). (B) The IQGAP1 overexpression improved the expression of β-catenin. Transfection with pCMV6 empty plasmids was taken as a control and the untreated HepG2 cells were taken as a mock. flag-IQ: pFlag-IQGAP1 plasmids. (C) The IQGAP1 knockdown by IQGAP1 siRNA (si-IQ) reduced β-catenin expression. Transfection with non-target siRNA was taken as a control (si-NC), and untreated HepG2 cells were taken as a mock. (D)The expression of IQGAP1 and β-catenin was gradually increased in several HCC cell lines. LO2 was a normal liver cell line. 97L: MHCC-97L; 97H: MHCC-97H.

We have found that the endogenous IQGAP1 interacts with β-catenin in HepG2 cells by co-IP analysis (Fig 1A). In order to verify the expression association between IQGAP1 and β-catenin, the protein expression changes of β-catenin was compared between IQGAP overexpression and knockdown in HepG2 cells. The β-catenin expression level was greatly increased to 3 times when IQGAP overexpression was up to 1.67 fold by transiently transfecting pFlag-IQGAP1 plasmids (Fig 1B). Consistently, about 50% IQGAP1 knockdown by IQGAP1-specific siRNA (si-IQGAP1) induced β-catenin downregulation to 78% (Fig 1C). IQGAP1 regulates β-catenin expression in HCC cells. Generally, either the endogenous or the exogenous expression of β-catenin can be regulated by IQGAP1 level.

For purpose of investigating the biological effects of the expression level of IQGAP1 and β-catenin in HCC progression, we further compared their expressions in several HCC cell lines with different metastasis abilities. As expected, IQGAP1 expression was gradually increased in HepG2, MHCC-97L and MHCC-97H cells than the normal liver cell line LO2 (Fig 1D). Especially it was greatly overexpressed in those with metastatic potentials, MHCC-97L and MHCC-97H cells. Corresponding to the higher expression of IQGAP1, β-catenin was respectively up-regulated to over 2, 3.2 and 4.8 folds among HepG2, MHCC-97L and MHCC-97H cells than LO2. There is an obviously positive correlation between the expression levels of the two proteins with HCC cell metastasis potentials.

### Overexpression of IQGAP1, β-catenin in HCC tissues and their association with HCC clinic characteristics

Furthermore, the associations of IQGAP1 and β-catenin expression were confirmed to play important roles in liver cancer progress from clinic immunohistochemistry analysis for liver cancer tissues as follows.

**Fig 2:**
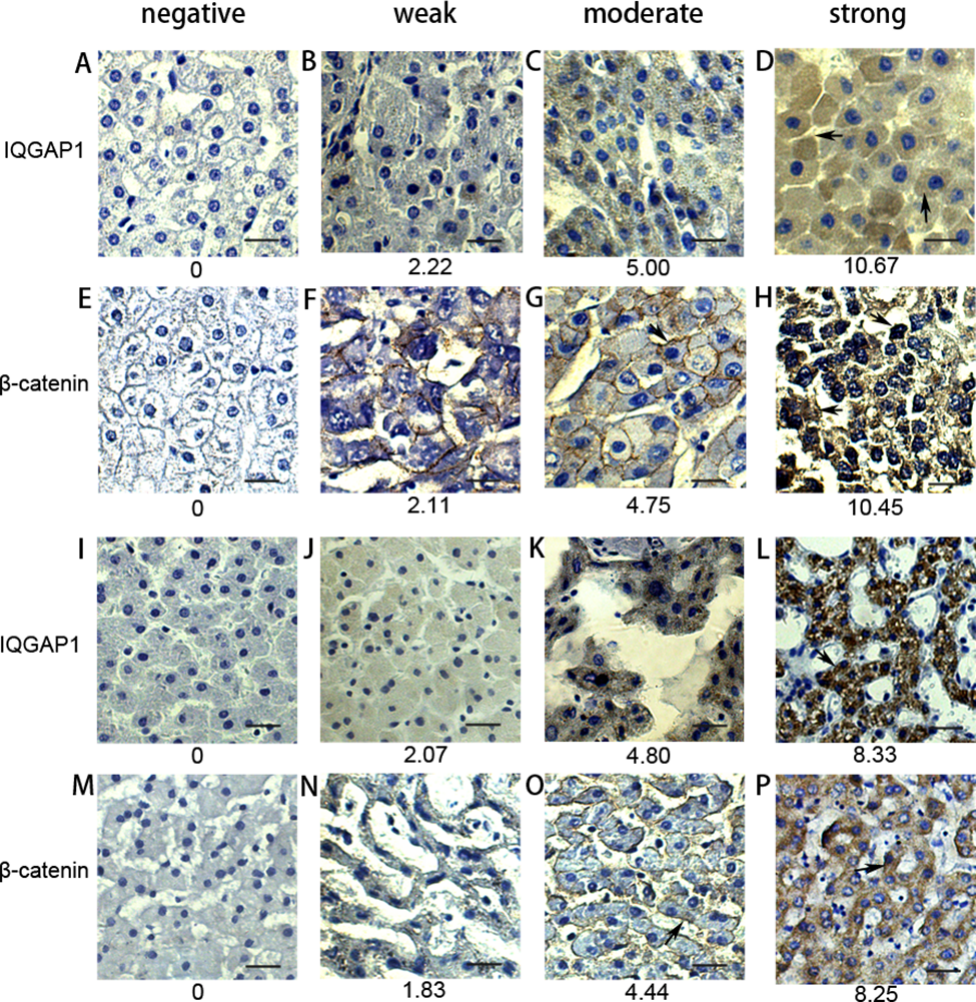
Different expression level of IQGAP1 and β-catenin in HCTs and PLTs. The negative, weak, moderate and strong staining activity of IQGAP1 was respectively shown in HCTs (A-D) and PLTs (I-L). IQGAP1 located in cytoplasm and cell membrane in HCTs (D), and IQGAP1 was observed in cytoplasm in PLTs (L). The β-catenin with negative, weak, moderate and strong staining activity was respectively detected in HCTs (E-H) and PLTs (M-P). The expression of β-catenin located in cell membrane (G), cytoplasm and nucleus (H) in HCTs, while it mainly located in cell membrane (O) and cytoplasm (P) in PLTs. Scale bar represents 50 μm (original magnification×400). HCTs: hepatocellular carcinoma tissues. PLTs: para-cancerous liver tissues.

The expression profiling of IQGAP1 and β-catenin *in vivo* were examined in 33 pairs of HCTs and patients’ autologous PLTs by immunostaining. Each tissue information and protein IHC scoring were summarized in the S1 and S2 Tables. The expression correlation of protein IQGAP1 and β-catenin was analyzed with a Spearman correlation. The association of these two proteins exhibited a significantly positive correlation of IQGAP1 *versus* β-catenin (Spearman r = 0.7030; *p*<0.001, n = 33). Out of all 33 cancer samples, IQGAP1 and β-catenin were overexpressed (strong expression) (Fig 2D and 2H) in 36.36% and 33.33% HCTs, averagely with immunoreactivity scoring over 10 (Table 1), which was far more than either the overexpression level or the percentage compared with the PLTs (p<0.05). Among the PLTs, IQGAP1 overexpression (Fig 2L) with 8.33 scores was detected in 9.09% samples, and β-catenin overexpression (Fig 2P) averagely with 8.25 scores was in 12.12% cases based on same scoring criteria. In 33 HCTs, except the non-expression (Fig 2A) and weak expression of IQGAP1 (Fig 2B), 8 cases (24.24%) showed moderate staining (Fig 2C), which was no significant difference with the 30.33% moderate expressing PLTs (Fig 2K). The expression distribution of IQGAP1 in HCTs was located in cytoplasm and cell membrane (Fig 2D). Our statistically significant data supported that IQGAP1 was obviously overexpressed in HCC.

Similarly, except 9 cases (27.27%) showed weak expression of β-catenin, the other 19 HCTs (57.57%) was obviously detected in expression, including 8 cases (24.24%) with moderate staining and 11 cases (33.33%) with overexpression in HCTs (Table 1). The expression distribution of β-catenin in HCTs was mainly located in cell membrane (Fig 2G), cytoplasm and nucleus (Fig 2H). While in PLTs, β-catenin was observed in cell membrane (Fig 2O) and cytoplasm (Fig 2P). Based on the same criteria, 25 cases (75.76%) of PLTs had β-catenin expression, including 36.36% (12 cases) weak expression, 27.27% (9 cases) moderate staining and 12.12% (4 cases) strong expression. And the other 8 PLTs showed no expression of β-catenin. For HCTs, 4 groups of negative, weak, moderate and strong expression were representatively showed in Fig 2E and 2H, and the corresponding staining in PLTs were showed in Fig 2M–2P. Generally, 11 cases (33.33%) of HCTs showed overexpression of β-catenin with average scores 10.45, whereas only 4 cases (12.12%) PLTs showed β-catenin overexpression with average scoring 8.25 (p<0.05).

**Table 1:** Protein immunoreactivity between HCTs and PLTs.

		HCCs (n = 33)			PLTs (n = 33)		
Protein	Immunoreactivity	Percentage	Total score	Average score	Percentage	Total score	Average score
IQGAP1	-	12.12% (4/33)	0	0	15.15% (5/33)	0	0
	+	27.27% (9/33)	20	2.22±0.22	45.45% (15/33)	31	2.07±0.21
	++	24.24% (8/33)	40	5.00±0.38	30.30% (10/33)	48	4.80±0.33
	+++	36.36% (n = 33)	128	10.67±0.48	9.09% (3/33)	25	8.33±0.33
β-catenin	-	15.15% (5/33)	0	0	24.24% (8/33)	0	0
	+	27.27% (9/33)	19	2.11±0.26	36.67% (12/33)	22	1.83±0.21
	++	24.24% (8/33)	38	4.75±0.37	27.27% (9/33)	40	4.44±0.29
	+++	33.33% (11/33)	115	10.45±0.55	12.12% (4/33)	33	8.25±0.25

* Student’s t test, p< 0.05.

HCTs: hepatocellular carcinoma tissues. PLTs: para-cancerous liver tissues.

Percentage: (specific case/total cases)

**-**: negative expression

**+**: weak expression

**++**: moderate expression

**+++**: Overexpression.

Furthermore, the association of protein expression with HCC clinic information was also analyzed. The clinicopathological characteristics of HCC samples included patients’ gender, age and tumor differentiation level. The average IHC score of IQGAP1 and β-catenin expression was 4.58 and 4.17 respectively, a moderate expression, among 24 HCC samples with a high differentiation (Table 2), while an overexpression level for IQGAP1 and β-catenin, with IHC scoring 8.67±0.99 and 8.00±1.26 respectively, exited in the other 9 HCC tissues with a low differentiation level (p<0.05). Therefore, the overexpression level of the two proteins, IQGAP1 and β-catenin, is associated with tumor low differentiation degree. On the other hand, the expression difference of the two proteins is neither related with patient’s age nor with human gender.

**Table 2:** The association of protein expression and HCC clinicopathological features.

Protein	Clinicopathologic variables	Case	Average score	Expression level
**IQGAP1**	Age (years)<50	17	5.12±0.99	++
	Age (years)>50	16	6.31±1.12	++
	High tumor differentiation	24	4.58±0.85	++
	Low tumor differentiation	9	8.67±0.99	+++
**β-catenin**	Age (years)<50	17	4.59±0.32	++
	Age (years)>50	16	5.88±1.31	++
	High tumor differentiation	24	4.17±0.81	++
	Low tumor differentiation	9	8.00±1.26	+++

* *P*<0.05, was considered statistically significant. *P*-value was calculated using Student’s t test.

**-**: negative expression

**+**: weak expression

**++**: moderate expression

**+++**: Overexpression.

### IQGAP1 prompts transcription and translocation expression of β-catenin

**Fig 3:**
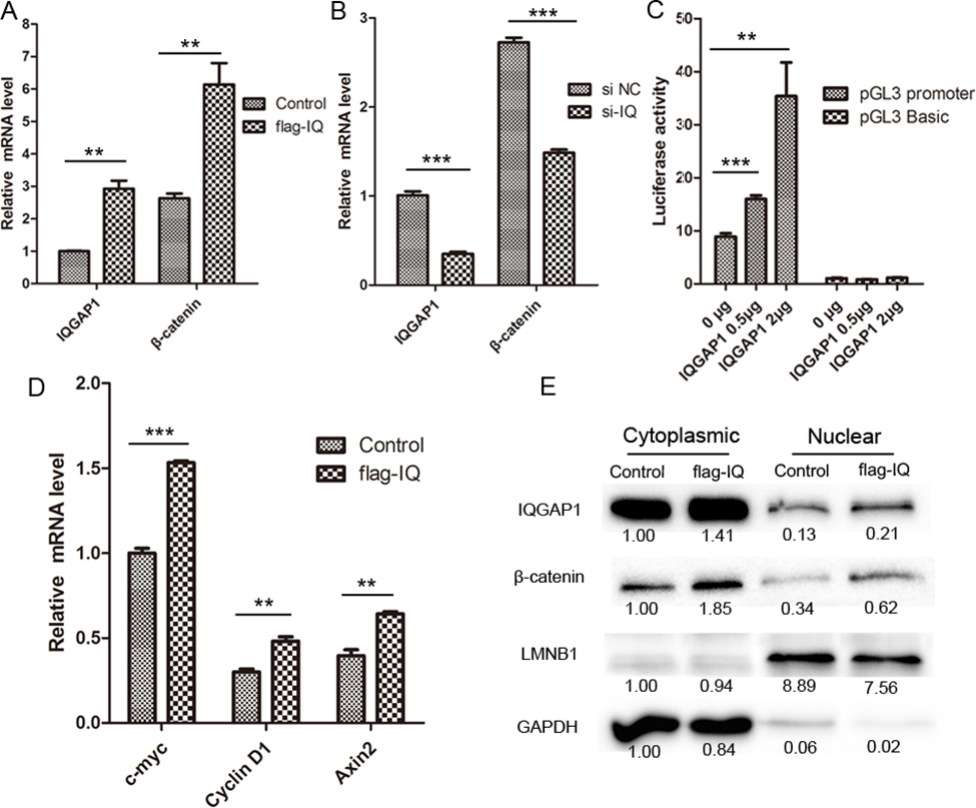
IQGAP1 activates β-catenin transcription and promotes its translocation to nucleus. (A) IQGAP1 overexpression by transfecting with pFlag-IQGAP1 plasmids (flag-IQ) upregulated β-catenin mRNA level. Transfection with empty pCMV6 plasmids was taken as a control. And the mRNA level of c-myc, cyclin D1 and Axin2 upon IQGAP1 overexpression was shown in (D). (B) IQGAP1 knockdown, by specific siRNA for IQGAP1 (si-IQ), decreased β-catenin mRNA level. Transfection with non-target siRNA was taken as a control (si NC). (C) IQGAP1 activates β-catenin transcription by a luciferase promoter analysis. (E) The overexpression of IQGAP1 induced β-catenin translocation from cytoplasm to nucleus. LMNB1 was a nuclear protein control. GAPDH was a cytoplasmic protein control. ** Student’s t test *p*<0.01, *** Student’s t test *p*<0.001.

Whether the regulation of IQGAP1 over beta-catenin is at the transcriptional or post-transcriptional level? Firstly we detected the mRNA level of IQGAP1 and β-catenin upon IQGAP1 overexpression and knockdown by real-time PCR. As results, when IQGAP1 mRNA was overexpressed to 2.9 times, the mRNA level of β-catenin was up-regulated to 2.34 times (Fig 3A). On the contrary, when IQGAP1 mRNA was knockdown to 35%, β-catenin mRNA was also decreased to 55% (Fig 3B). So far IQGAP1 can regulate β-catenin at the mRNA level. Furthermore, a luciferase β-catenin promoter analysis was consistent with the transcriptional regulation. As shown in the Fig 3C, when HepG2 cells were co-transfected with 1μg pGL3-catenin plasmids and 0.5μg pFlag-IQGAP1, the luciferase activity exhibited 1.8 times than control cells which were co-transfected with 1 μg pGL3-catenin plasmids and 0.5μg pCMV empty plasmids. And when HepG2 cells were co-transfected with 1μg pGL3-catenin plasmids and 2 μg pFlag-IQGAP1, the luciferase activity was increased to 3.98 times than control cells which were co-transfected with 1 μg pGL3-catenin plasmids and 2μg pCMV empty plasmids. In addition, the luciferase activity had no obvious changes in two groups of control cells co-transfected with 1μg pGL3 empty plasmids and 0.5μg or 2 μg pFlag-IQGAP1 plasmids. These results indicated IQGAP1 can activate β-catenin transcription.

Next, we evaluated whether the upregulated β-catenin induced some target gene changes upon IQGAP1 overexpression. Response for regulations of β-catenin and IQGAP1, the mRNA expression level of three target genes, including c-myc, cyclin D1 and Axin2 were measured by real-time PCR [21]. The upregulation of beta-catenin induced a higher expression of its direct target genes (Fig 3D). Response for a 1.34-fold upregulation of β-catenin mRNA in HepG2 cells transiently transfected with pFlag-IQGAP1 plasmids (Fig 3A), the mRNA level of c-myc, cyclin D1 and Axin2 was respectively increased to 1.53, 1.60 and 1.62 folds (Fig 3D). Furthermore, in order to determine cellular distribution changes of IQGAP1 and β-catenin, we separately detected protein expression profiling in cytoplasm and nuclear through protein subfractionation analysis. Besides an increase of cytoplasmic β-catenin upon IQGAP1 overexpression, nuclear IQGAP1 and beta-catenin was respectively up-regulated to 1.92 and 2.12 times in IQGAP1-overexpressing cells (Fig 3E), which indicated IQGAP1 upregulation improved the transcription and expression of β-catenin, and the increased β-catenin could translocated to the nucleus.

### Upregulation of IQGAP1 and β-catenin regulates cell proliferation and migration

Based on our findings, IQGAP1 and β-catenin are usually overexpressed *in vitro* and *in vivo*, and their overexpression levels are associated with tumor malignancy degree of HCC. Therefore we hypothesized that IQGAP1 may play a role in mediating cell proliferation and cell migration.

**Fig 4:**
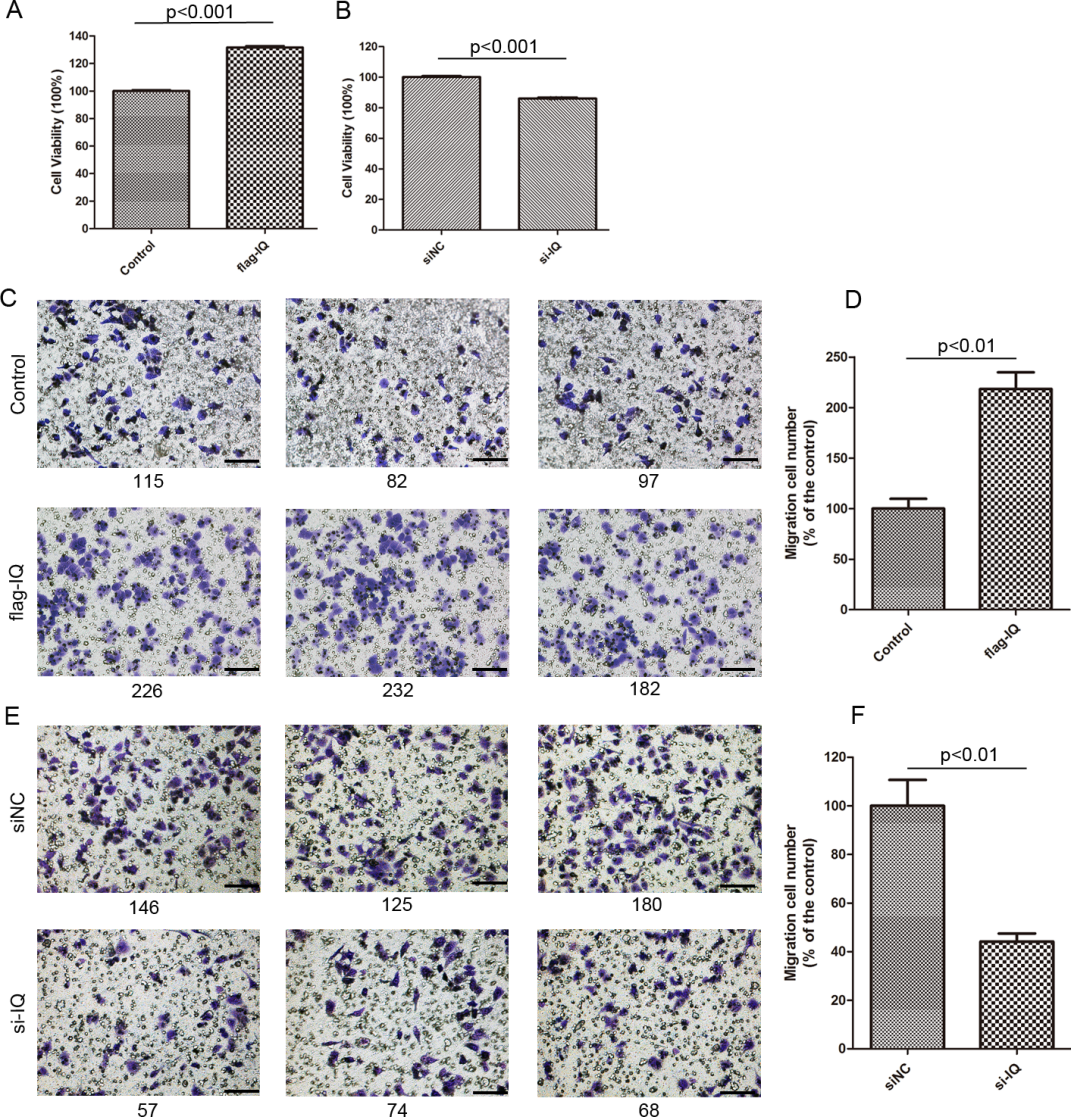
IQGAP1 regulates cell proliferation and migration ability. Overexpression of IQGAP1 in HepG2 cells enhanced cell proliferation (A) and cell migration (C, D). Knockdown of IQGAP1 decreased cell growth (B) and migration (E, F). Control: HepG2 cells transfected with empty pCMV6 plasmids; flag-IQ:HepG2 cells transfected with pFlag-IQGAP1 plasmids. siNC: HepG2 cells transfected with control siRNA; si-IQ: HepG2 cells transfected with IQGAP1 siRNA. Scale bar represents 100 μm (original magnification×200).

Response to IQGAP-overexpression (Fig 1B) and knockdown (Fig 1C), cell proliferation was significantly increased to 132% or decreased to 86% (n = 3, *p*<0.001) in cell number (Fig 4A and 4B). Meanwhile cell migration ability was also assayed using a transwell-cultured chamber. Cells transiently transfected with pFlag-IQGAP1 plasmids exhibited a mean 2.18-fold of cell migration numbers compared to that transfected with empty plasmids (n = 3, *p*<0.01) (Fig 4C and 4D). Whereas the knockdown of IQGAP1 by IQGAP1-specific siRNA in HepG2 cells showed a low migration ability compared with the mock group cells transfected with non-target control oligonucleotides at a ratio of 0.44 (n = 3, *p*<0.01) (Fig 4E and 4F).

Moreover we repeated the experiments in another HCC cell line HuH7, and the results also indicated high expression level of IQGAP1 promoted cell proliferation and migration ability (S1 Fig). The conclusion was same as the data from HepG2 cells (Fig 4). In IQGAP1-overexpressing HuH7 cells, cell proliferation was significantly increased to 125% (n = 3, *p*<0.001) approximately detected by MTT, and cell migration ability was enhanced to 1.33-fold compared to the transfected with empty plasmids (n = 3, *p*<0.01). On the contrary, when IQGAP1 knockdown by IQGAP1-specific siRNA in HuH7 cells, cell growth was decreased to 79% (n = 3, *p*<0.01) in cell quantity, and a lower cell migration with 0.70-fold downregulation was observed (n = 3, *p*<0.05) compared with mock cells transfected with non-target control oligonucleotides.

Flow cytometry analysis was performed to eliminate the decreased cell proliferation effects caused by cell death (S2 Fig.). Corresponding to the IQGAP1 knockdown by IQGAP1-specific siRNA in the Fig 4, after HepG2 cells were transfected with IQGAP1-specific siRNA for 48h, we harvested cells and stained with Annexin V-FITC/PI to determine if the proliferation rate was decreased due to cell death. The cell death ratio was 5.17% ± 0.42%, 6.78% ± 0.50% respectively for cells transfected with non-target control oligonucleotides (si NC) and those transfected with IQGAP1-specific siRNA. It was shown cell death had no significant differences after IQGAP1 knockdown (*p*>0.05), which indicated that the observed effects are only due to changes in proliferation rate.

**Fig 5:**
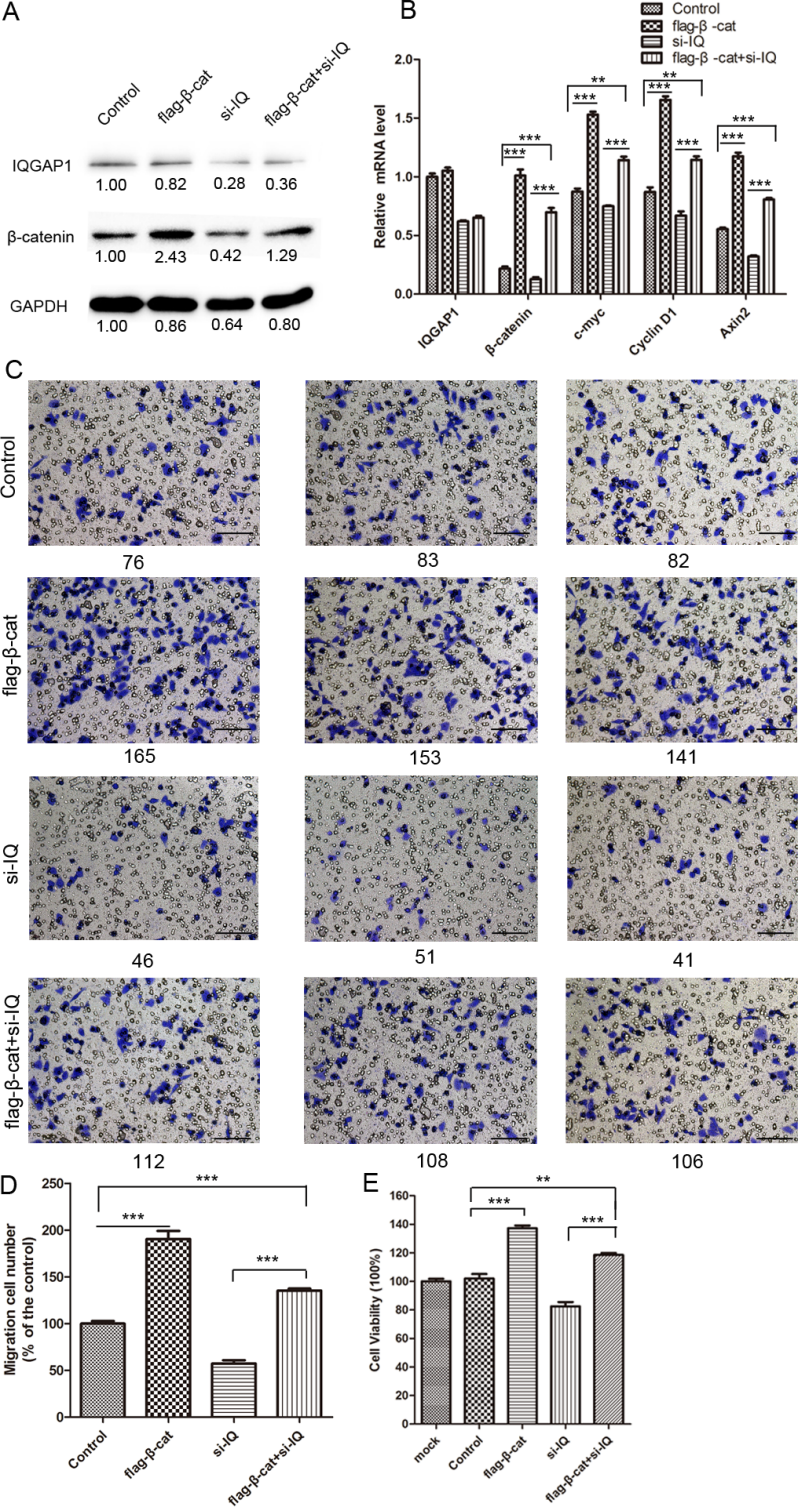
β-catenin regulates cell growth and migration. (A) β-catenin mRNA exhibited a recovery increase in IQGAP1-knockdown HepG2 cells re-transfected β-catenin plasmids. The upregulation of β-catenin induced the mRNA expression of c-myc, cyclin D1 and Axin2 (B), and enhanced cell proliferation (E) and migration (C, D). Control: HepG2 cells transfected with empty vectors. flag-β-cat: HepG2 cells transfected with pFlag-β-catenin plasmids. si-IQ: HepG2 cells transfected with IQGAP1 siRNA; flag-β-cat+si-IQ: HepG2 cells transfected with pFlag-β-catenin plasmids on the condition of IQGAP1 knockdown. ** Student’s t test *p*<0.01, *** Student’s t test *p*<0.001. Scale bar: 100 μm (original magnification×200).

In order to investigate the functional associations of IQGAP1 with β-catenin, a rescue experiment was performed by overexpressing β-catenin in the condition of IQGAP1 knockdown. When the endogenous IQGAP1 in HepG2 was greatly inhibited by IQGAP1 siRNA (Fig 5A, lane 3, the upper), the downregulation of β-catenin was induced both at mRNA (Fig 5B) and protein level (Fig 5A, lane 3, the middle), following the targeting genes (c-myc, cyclin D1 and Axin2) were reduced at mRNA level (Fig 5B). Furthermore, cell growth and migration was also inhibited to 57.3% and 82.6% (Fig 5C) compared to the control. On the other hand, compared with IQGAP1 knockdown, the overexpressing β-catenin under IQGAP1 knockdown resulted in a recovery increase of the downstream genes, and cell proliferation and migration was obviously upregulated too. The β-catenin protein level was almost 2.5 times than that in the IQGAP1 knockdown group and the mRNA level was increased to 5.45-fold. As expected, the c-myc, cyclin D1 and Axin2 mRNA level was increased to 1.52, 1.71 and 2.51 times compared with IQGAP1-knockdown group, so as cell migration was increased to 1.35 times and proliferation ability was increased to 143%. These results indicated that the alteration of β-catenin level is due to the gain or loss of IQGAP1 expression, in which their expressions and associations have effects on cell growth and migration.

In combination with the protein expression level changes of IQGAP1 and β-catenin and cell growth, we can conclude that the overexpression of IQGAP1 and β-catenin *in vitro* and *in vivo* promotes cell proliferation and migration ability in HCC, while their downregulation reduces cell growth and migration.

### IQGAP1 and β-catenin interacting network discovered by bioinformatics analysis

**Fig 6:**
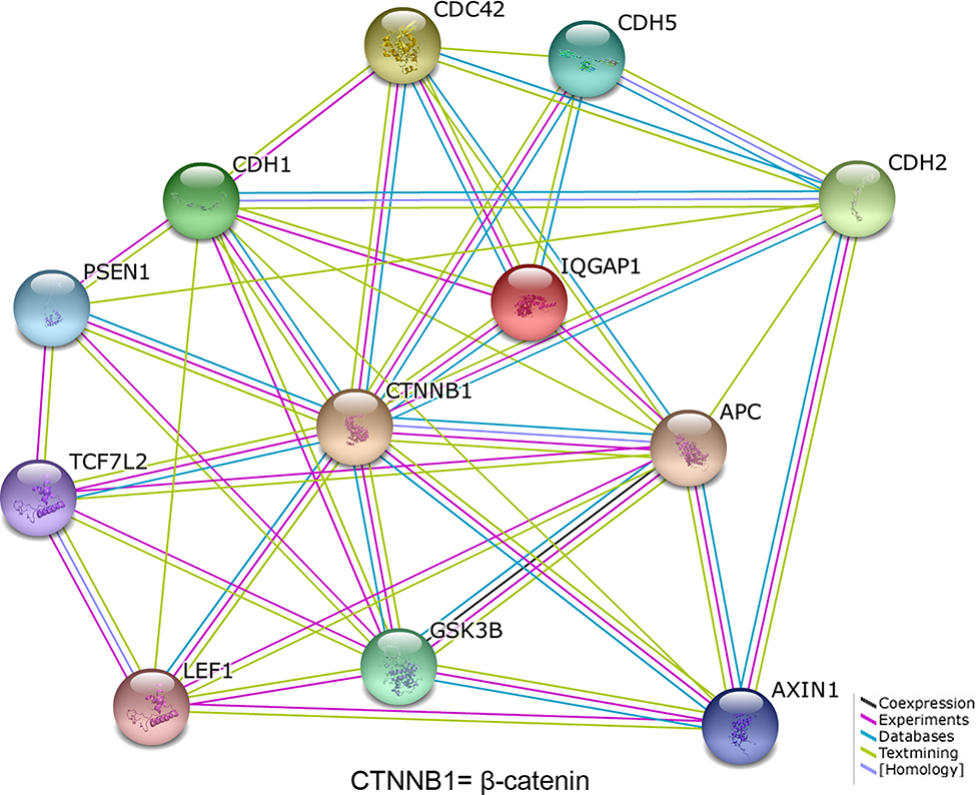
The interacting proteins with IQGAP1 and β-catenin analyzed by a bioinformatics software STRING. The functional partners were predicted by different methods, which were shown in different line colors. black (\, coexpression), means genes co-expressed in same or in other species; purple (\, experiments), shows a significant protein interaction from literatures; light blue (\, database), shows a significant protein interaction group gathered from curated databases; yellow (\, textmining), shows protein interaction groups extracted from scientific literatures; grey blue (\, homology), shows a protein interaction group gathered from homology. The predicted functional partners include as follows. APC, adenomatous polyposis coli; CDC42, cell division cycle 42; CDH5, cadherin 5; CDH2, cadherin 2, type 1, N-cadherin; CDH1, cadherin 1, type 1, E-cadherin; AXIN1, axin 1; GSK3B, glycogen synthase kinase 3 beta; LEF1, lymphoid enhancer-binding factor 1; PSEN1, presenilin 1; TCF7L2, transcription factor 7-like 2.

Due to the multiple binding partners of IQGAP1 (Fig 6) based on the online software STRING, it has been indicated that IQGAP1 lies in the central position to interact with different proteins, including β-catenin, cell division cycle 42 (CDC42), E-cadherin (CDH1) and adenomatous polyposis coli (APC) to promotes cell motility and invasion. In the protein interaction map, several proteins, including CDC42, E-cadherin and APC dynamically involve in the interactions with IQGAP1 and β-catenin. For example, the activated CDC42 positively regulates E-cadherin-mediated cell-cell adhesion by inhibiting the interaction of IQGAP1 with β-catenin[22]. The different ratio of E-cadherin–β-catenin–IQGAP1 complex to E-cadherin–β-catenin–α-catenin complex would result in different adhesion type and cell-cell dissociation[3]. Under these conditions, IQGAP1 does not bind to β-catenin and cannot dissociate α-catenin from the cadherin-catenin complex, leading to strong adhesion. By contrast, IQGAP1 is freed from CDC42/Rac1 complex and interacts with β-catenin to dissociate α-catenin from the cadherin-catenin complex, which results in weak adhesion and promotes cell migration[4]. The β-catenin, APC, GSK3B, AXIN1, LEF1, and TCF7L2 are all parts of the WNT signaling [23]. And IQGAP1 is reported to take part in WNT signaling pathway [24]. So far, we estimate that IQGAP1 interacts with β-catenin to take part in WNT signaling pathway to regulate cell proliferation and cell migration.

## Discussion

In eukaryotic cells, scaffold proteins play crucial roles in many important signaling pathways [25, 26]. As a scaffold protein, IQGAP1 could interact with a number of proteins which could lead to oncogenesis. The alteration of IQGAP1 expression and localization correlate with cancer progression in several human primary tumors [5, 27–30]. Our studies discovered that IQGAP1 interacts with β-catenin, and both of their overexpression level regulates cell proliferation and cell migration in HCC.

We have demonstrated that the overexpression of IQGAP1 can upregulate the expression of β-catenin. In several hepatocellular cell lines, the overexpression level of IQGAP1 and its binding protein β-catenin have a positive correlation with cell metastasis potentials due to their contributions for cell proliferation and migration. And a significantly higher expression of IQGAP1 and β-catenin also usually exists in human HCC tissues; especially their overexpression is clinically correlated with tumor malignancy or differentiation degree. The aberrant accumulation of β-catenin is observed at high frequency in many cancers [31]. This accumulation correlates with either mutational activation of β-catenin or mutational inactivation of APC and Axin1 genes in some tumors [32, 33]. However, not all the β-catenin accumulation contacted with the absence of a mutation in these genes[34]. Thus, there must be additional sources for aberrant β-catenin accumulation in cancer cells. Here, we confirmed that the overexpression of β-catenin is regulated by IQGAP1 to promote cell growth and migration in HCC.

Due to multiple interacting partners of IQGAP1 (Fig 6), IQGAP1 and its interacting protein β-catenin can involve in different signal pathways to regulate cell proliferation and mobility. β-catenin plays an important role in cell-cell adhesion at the plasma membrane and in transactivation of specific genes via TCF/LEF transcription factors in the nucleus[35]. In addition, the nuclear accumulation of β-catenin can also promote cell migration and cell transformation [36, 37]. In our study, the cellular distribution of β-catenin is partially shifted to nucleus in HCC samples (Fig 2H) compared with the normal PLTs, and the overexpression of IQGAP1 promotes β-catenin translocation to nucleus in HepG2 cells (Fig 3E), which both are consistent with the previous reports. In this paper, we discovered that IQGAP1 can regulate β-catenin expression and nuclear accumulation, meanwhile their upregulation and association induce β-catenin-targeting gene expression and promote cell migration.

## Conclusions

The overexpression of IQGAP1 and β-catenin is associated with tumor progression in HCC *in vitro* and *in vivo* due to their contributions for cell proliferation and migration.

## Supporting Information

S1 Table Protein IHC scoring for hepatocellular carcinoma tissues. (XLSX)

S2 Table Protein IHC scoring for para-cancerous liver tissues. (XLSX)

S1 Fig IQGAP1 regulated cell proliferation and migration of HuH7 cells. Cells were stained with Annexin V-FITC/PI. siNC: HepG2 cells transfected with control siRNA; si-IQ: HepG2 cells transfected with IQGAP1 siRNA.
(TIF)

S2 Fig Cell death detected by flow cytometry. IQGAP1 regulated cell proliferation and migration ability of HuH7 cells. Overexpression of IQGAP1 enhanced cell proliferation (A) and cell migration (C, D) in HuH7 cells. IQGAP1 Knockdown decreased cell proliferation (B) and migration (E, F). Control: HuH7 cells transfected with empty pCMV6 plasmids; flag-IQ: HuH7 cells transfected with pFlag-IQGAP1. siNC: HuH7 cells transfected with control siRNA; si-IQ: HuH7 cells transfected with IQGAP1 siRNA. Scale bar represents 100 μm (original magnification×200).
(TIF)
