# Supplementary figures and images for: The Overexpression of IQGAP1 and β-Catenin Is Associated with Tumor Progression in Hepatocellular Carcinoma In Vitro and In Vivo
Source: PLoS One. 2015 Aug 7;10(8):e0133770. doi: 10.1371/journal.pone.0133770 (PMC4529304; doi:10.1371/journal.pone.0133770)

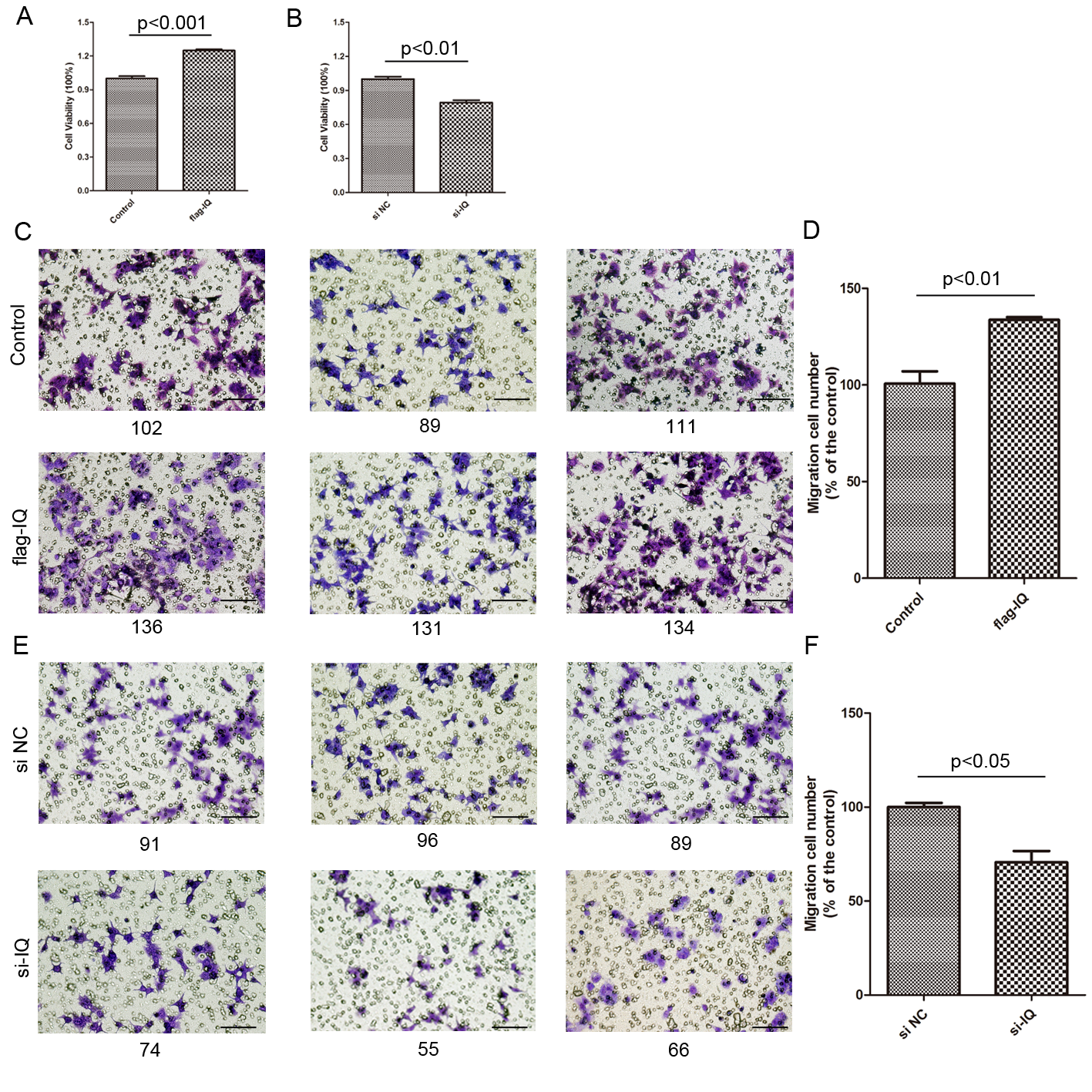

Supplement: S1 Fig — Cells were stained with Annexin V-FITC/PI. siNC: HepG2 cells transfected with control siRNA; si-IQ: HepG2 cells transfected with IQGAP1 siRNA. (TIF) [file pone.0133770.s003.tif]

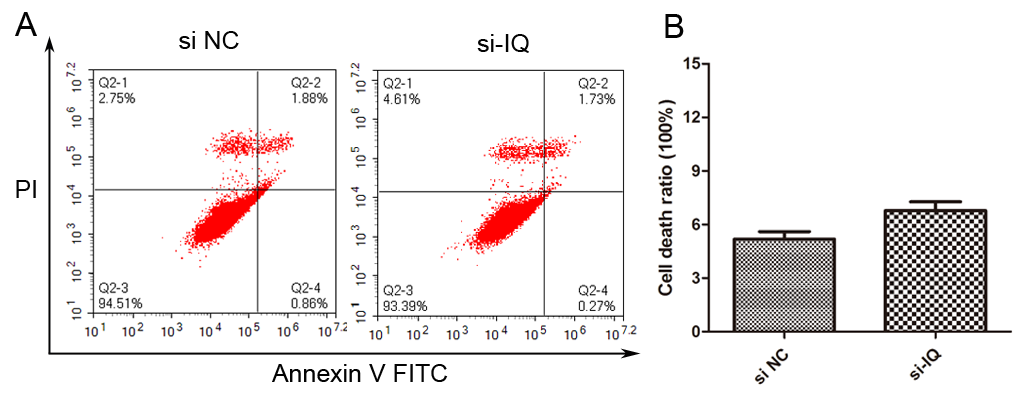

Supplement: S2 Fig — IQGAP1 regulated cell proliferation and migration ability of HuH7 cells. Overexpression of IQGAP1 enhanced cell proliferation (A) and cell migration (C, D) in HuH7 cells. IQGAP1 Knockdown decreased cell proliferation (B) and migration (E, F). Control: HuH7 cells transfected with empty pCMV6 plasmids; flag-IQ: HuH7 cells transfected with pFlag-IQGAP1. siNC: HuH7 cells transfected with control siRNA; si-IQ: HuH7 cells transfected with IQGAP1 siRNA. Scale bar represents 100 μm (original magnification×200). (TIF) [file pone.0133770.s004.tif]
